# Melatonin Reverses Fas, E2F-1 and Endoplasmic Reticulum Stress Mediated Apoptosis and Dysregulation of Autophagy Induced by the Herbicide Atrazine in Murine Splenocytes

**DOI:** 10.1371/journal.pone.0108602

**Published:** 2014-09-26

**Authors:** Shweta Sharma, Jayanta Sarkar, Chandana Haldar, Sudhir Sinha

**Affiliations:** 1 Biochemistry Division, CSIR-Central Drug Research Institute, Lucknow, Uttar Pradesh, India; 2 Department of Zoology, Banaras Hindu University, Varanasi, Uttar Pradesh, India; 3 Academy of Scientific and Innovative Research, New Delhi, India; National Cheng Kung University, Taiwan

## Abstract

Exposure to the herbicide Atrazine (ATR) can cause immunotoxicity, apart from other adverse consequences for animal and human health. We aimed at elucidating the apoptotic mechanisms involved in immunotoxicity of ATR and their attenuation by Melatonin (MEL). Young Swiss mice were divided into control, ATR and MEL+ATR groups based on daily (x14) intraperitoneal administration of the vehicle (normal saline), ATR (100 mg/kg body weight) and MEL (20 mg/kg body weight) with ATR. Isolated splenocytes were processed for detection of apoptosis by Annexin V-FITC and TUNEL assays, and endoplasmic reticulum (ER) stress by immunostaining. Key proteins involved in apoptosis, ER stress and autophagy were quantified by immunoblotting. ATR treatment resulted in Fas-mediated activation of caspases 8 and 3 and inactivation of PARP1 which were inhibited significantly by co-treatment with MEL. MEL also attenuated the ATR-induced, p53 independent mitochondrial apoptosis through upregulation of E2F-1 and PUMA and suppression of their downstream target Bax. An excessive ER stress triggered by ATR through overexpression of ATF-6α, spliced XBP-1, CREB-2 and GADD153 signals was reversed by MEL. MEL also reversed the ATR-induced impairment of autophagy which was indicated by a decline in BECN-1, along with significant enhancement in LC3B-II and p62 expressions. Induction of mitochondrial apoptosis, ER stress and autophagy dysregulation provide a new insight into the mechanism of ATR immunotoxicity. The cytoprotective role of MEL, on the other hand, was defined by attenuation of ER stress, Fas-mediated and p53 independent mitochondria-mediated apoptosis as well as autophagy signals.

## Introduction

Environmental or occupational exposure to the herbicide Atrazine (ATR, 6-chloro-N^2^-ethyl-N^4^-isopropyl-1,3,5-triazine-2,4-diamine), which is one of the most commonly used agricultural products worldwide, can produce toxic consequences in animals and humans [1]–[3]. As a potent endocrine disruptor, ATR targets endocrine, reproductive and nervous systems [4]–[6]. In addition, it also modulates immunity [7] due to the interaction between endocrine and immune systems [8]. ATR-induced suppression of immunity foreshadows the risk for contracting diseases. Indeed, some ATR applicators have been found to develop chronic bronchitis accompanied by a modulation of their humoral and cellular immune responses [9]. In mice, ATR induced immunotoxicity is manifested by a decline in the number of splenic dendritic cells, selected subpopulations of splenic lymphocytes, thymic T cell populations and Natural Killer cell activity along with an increase in early apoptosis and reduction in weights of spleen and thymus [2], [10]–[12]. To date, Fas-mediated apoptosis has been identified as a mechanism for ATR immunotoxicity in splenocytes of mice [13] though additional mechanisms for immunotoxicity may also exist.

Endoplasmic reticulum (ER) plays a key role in synthesis and maturation of proteins, biosynthesis of lipids, regulation of calcium (Ca^2+^) and maintenance of cell homeostasis. Disturbance in any of these functions activates the ‘ER stress pathway’ comprising three different branches: activating transcription factor 6 (ATF6), inositol-requiring enzyme 1 (IRE1) and protein kinase RNA-like ER kinase (PERK); which involve activation of transcription factors ATF-6α, X-box binding protein-1 spliced form (XBP-1s), cAMP-response element binding protein-2 (CREB-2) and growth arrest and DNA damage inducible 153 (GADD153). Activation of ER stress pathway is an adaptive response to restore ER homeostasis. However, a prolonged ER stress triggers apoptosis [14] which can contribute to pathophysiological conditions such as neurodegenerative diseases and diabetes [15], [16]. Autophagy, a dynamic catabolic process that degrades misfolded proteins, organelles and other cytoplasmic contents, serves as a survival mechanism against ER stress [17]. ER stress and autophagy dysregulation have been linked with toxicity induced by certain pesticides and herbicides [18]. However, it is not known whether ATR can also modulate the ER stress and autophagy pathways.

Melatonin (MEL, *N*-acetyl-5-methoxytryptamine) is a ubiquitous indoleamine hormone produced by the pineal gland, retina, gastrointestinal tract, Harderian gland, bone marrow, platelets, lymphocytes, skin, thymus and spleen [19]. Its amphiphilic nature determines its bioavailability as well as action on cells and sub-cellular organelles [20]. The multifunctional versatility of MEL as a circadian rhythm regulator, antioxidant, free radical scavenger as well as an antiapoptotic (for normal cells), oncostatic and immunomodulatory agent has led to it being considered as a molecule with immense therapeutic potential [21], [22]. MEL reduces ER stress and apoptosis caused by the herbicide arsenite by repressing the activation of XBP-1 (IRE-1 branch) and caspase-3 [23]. It is not however clear whether it can regulate all three branches of the ER stress pathway in response to the action of herbicides. Role of MEL in protecting normal cells from apoptosis through modulation of Fas-mediated pathway also remains unresolved [24]. It also remains debatable whether MEL is an inhibitor or stimulator of autophagy [25], [26].

This study was aimed at elucidating the mechanism of ATR immunotoxicity in mice and its amelioration by MEL. Since apoptosis, ER stress and autophagy are interrelated, modulation of any of these processes could contribute to immunotoxicity. We therefore monitored changes in expression levels of the key apoptotic, ER stress and autophagic signals in splenocytes in response to administration of ATR, with or without MEL. We found that MEL ameliorated ATR-induced cell death in mouse splenocytes through suppression of Fas, E2F-1 and ER stress mediated apoptosis and also through restoration of impaired autophagy. These findings extend our knowledge of health risks involved in exposure to herbicides such as ATR as well as the therapeutic potential of MEL, particularly against immunotoxicity.

## Materials and Methods

### Reagents

MEL, ATR, RPMI-1640, fetal bovine serum (FBS), dimethylsulfoxide (DMSO), ammonium chloride (NH_4_Cl), bovine serum albumin (BSA), poly-L-lysine, Tween-20, Tris buffer, 30% acrylamide/bis-acrylamide solution, Annexin V-FITC kit, β-actin and HRP-conjugated secondary antibodies were obtained from Sigma-Aldrich (St. Louis, MO, USA). Alexa Fluor 594 antibody and Prolong Gold antifade reagent with DAPI were procured from Invitrogen (Carlsbad, CA, USA). Polyvinylidene fluoride (PVDF) membrane and Immobilon Western chemiluminiscent substrate were purchased from Millipore (Bedford, MA, USA). Mammalian protein extraction reagent (M-PER) and Bichinchonoic acid (BCA) protein assay kit were procured from Pierce Biotechnology (Rockford, IL, USA). DeadEnd Fluorometric TUNEL kit was purchased from Promega (Madison, WI, USA).

### Ethics statement

All experimental procedures on mice were approved by the Institutional Animal Ethics Committee (IAEC) of CSIR-Central Drug Research Institute (Permit No. 34/1999/CPCSEA). All ethical guidelines and animal welfare regulations set out by IAEC and Committee for the Purpose of Control and Supervision of Experiments on Animals (CPCSEA), Government of India were strictly followed. Mice were sacrificed by cervical dislocation.

### Animals

Swiss mouse is widely used as a model for toxicological or pharmacological evaluation of drugs. *In vivo* protective effects of MEL have also been demonstrated in these mice [27], [28]. Young male Swiss albino mice (aged 4 weeks, weighing 20±2 g) used in this study were bred and reared at National Laboratory Animal Centre, CSIR-CDRI. They were housed in a controlled environment (22–26°C; 50–60% humidity; 12 h light/dark cycle) and fed standard rodent food pellet and water *ad libitum*. After 10 days of acclimatization, animals were subjected to the planned treatments and monitored daily for behavioural change, food and water intake, body weight and survival.

### Treatments

Taking a cue from earlier reports [10], [13], [29], the experimental animals were treated intraperitoneally (i.p.) with ATR (100 mg/kg body weight/day), with or without MEL (20 mg/kg b.wt./day) for 14 days. Comparable ATR treatment schedules have been reported to show a significant modulation of immunological parameters, including apoptosis, in splenocytes of young mice [10], [13]. Similarly, co-treatment with an identical dose of MEL has been shown to produce significant reduction in the oxidative damage induced by a diquat herbicide in murine splenocytes [29]. ATR stock was made in DMSO and diluted with 0.9% saline to obtain the required dosage whereas MEL was dissolved in minimal amount of ethanol before diluting with saline. Stock solutions were stored at 4°C for one week and working dilutions were prepared fresh daily before use. Animals were randomly divided into 3 groups of 6 mice each. Control (CON) group received the vehicle (0.9% saline, i.p.), ATR group received ATR alone and MEL+ATR group was administered MEL 30 min before ATR. The levels of endogenous MEL and total antioxidants are lowest during the daytime [19] favouring enhanced oxidative damage by toxins [30]. Therefore, treatments were given during daytime, at around 11∶00 am. All end points were studied at 24 h after the last treatment. After recording body weights, animals were sacrificed by cervical dislocation and organs were excised aseptically. Weights of spleen and thymus were noted immediately.

### Relative organ weight

Relative organ weight was determined as the ratio of weight of the organ (mg) to body weight (g) of a mouse.

### Preparation of splenocytes

Spleen was placed on a cell strainer (70 µm pore size) in a petridish containing RPMI-1640 medium (complete with 10% FBS) and pressed gently with the plunger of glass syringe to disperse cells into the medium. Erythrocytes were lysed by hypotonic shock with equal volume of cold 0.84% Tris-NH_4_Cl (pH 7.2). The splenocytes were washed, resuspended in complete RPMI medium and counted in a Neubauer’s counting chamber.

### Annexin V- fluorescein isothiocyanate (FITC)/propidium iodide (PI) double staining assay

Apoptotic cells were detected using Annexin V-FITC kit. Splenocytes (10^6^ cells/mL) were resuspended in binding buffer and incubated in dark for 10 min at room temperature (RT) with Annexin V-FITC conjugate and PI. Population of unstained splenocytes were gated to locate quadrant using FACS Calibur flow cytometer (Becton-Dickinson, CA, USA). Later, fluorochrome labelled cells were analysed for FITC+/PI- apoptotic events. The percentage of apoptotic cells (of the total counted cells) was expressed as apoptotic index.

### Terminal deoxynucleotidyl transferase mediated dUTP nick end labeling (TUNEL) assay

DNA fragmentation in apoptotic cells was detected using fluorometric TUNEL kit. Briefly, cells were fixed with 4% paraformaldehyde, washed, spotted onto poly-L-lysine coated slides and air-dried. Cells were permeabilized with 0.2% Triton X-100, washed and covered with equilibration buffer. Later, rTdT incubation buffer (nucleotide mix and rTdT enzyme in equilibration buffer) was added on to cells and slides were kept in a humidified chamber in dark (37°C, 1 h). Reaction was stopped by adding 2x SSC reagent. After washing, cells were counterstained with freshly diluted PI (1 µg/mL), which stains apoptotic as well as non-apoptotic cell nuclei. Apoptotic cells displaying green fluorescence (due to catalytic incorporation of fluorescein-12-dUTP at 3′OH DNA ends) were determined as TUNEL positive. Over 200 nuclei were counted in a randomly selected field, using Nikon Eclipse Ti inverted microscope (Nikon Instruments, NY, USA) at 200× magnification. The percentage of TUNEL positive nuclei (of the total number of nuclei) was expressed as TUNEL positivity index.

### Immunofluorescence

Cells were resuspended in PBS and fixed with equal volume of 4% paraformaldehyde (RT, 15 min). The washed cells were spotted onto poly-L-lysine coated cover glass and air-dried. Cells were permeabilized (4°C, 15 min) with ice-cold 0.3% Triton X-100 in PBS, washed with PBS containing 0.1% Tween-20 (PBST) and incubated (RT, 1 h) in blocking solution (2% BSA in PBST). Subsequently, cells were incubated (37°C, 1 h, in humidified atmosphere) with primary antibodies against ATF-6α, XBP-1 and GADD153 proteins (Table S1). Cells were later washed with PBST, incubated (37°C, 1 h) with Alexa-Fluor 594 secondary antibody (1∶300) and washed again with PBST. Cover slips were mounted on antifade reagent (∼10 µL). After overnight incubation in dark, cells were observed for expression of signals under Nikon Eclipse Ti microscope (200× magnification).

### Preparation of cell lysates

Cell suspension was washed, resuspended in PBS and centrifuged (300 *g*×10 min, 4°C). Cell sediments were lysed (30 min, on ice) with MPER reagent (containing protease inhibitor cocktail) to extract total protein. Lysates were centrifuged (14000 *g*×10 min, 4°C) and supernatants collected. After estimating protein by BCA kit, samples were stored in aliquots at −80°C.

### Immunoblotting

Samples (20 µg) were subjected to SDS-PAGE on 8% and 12% resolving gels and proteins were transferred electrophoretically onto PVDF membrane. The membrane was blocked with 5% non-fat dry milk in TBST {Tris-buffered saline (10 mM Tris, 100 mM NaCl), containing 0.1% Tween-20} and incubated overnight (4°C) with primary antibodies (FasL, Fas, caspases 8, 3, PARP1, p53, E2F1, PUMA, ATF6, XBP1, CREB2, GADD153, BECN1, LC3B, p62) using indicated dilutions (Table S1). After washing with TBST three times for 5 min each, the membranes were incubated with HRP-conjugated secondary antibody (1∶8000, 1 h, RT). After another wash cycle, the membranes were developed using ECL reagent and visualized in ChemiDoc (Bio-Rad Laboratories, USA). Densities of specific bands were quantified using Image J analysis software (version 1.46, NIH, USA). Density of each band was normalized by that of the loading control (β-actin).

### Statistical analysis

All data are expressed as mean ± standard error of mean (SEM) of at least 3 independent experiments. Statistical significance was determined using one way ANOVA followed by Student’s Newman-Keuls *post hoc* test. *P*<0.05 was considered as statistically significant.

## Results

### Relative weight loss of lymphoid organs due to ATR treatment was reversed by MEL

We initially measured relative weights of spleen and thymus as a gross sign of immunotoxicity in response to ATR treatment. The 14-day treatment schedule resulted in a significant reduction of relative weights of both organs, with spleen showing greater susceptibility to treatment (*P*<0.01 versus CON; Figure S1). This observation is corroborated by a previous report [10]. Co-treatment with MEL resulted in significant recovery of relative weights of spleen and thymus (*P*<0.05 versus ATR; Figure S1), indicating ameliorating effect of MEL on ATR immunotoxicity.

### Apoptosis in splenocytes induced by ATR was inhibited by MEL

**Figure 1:**
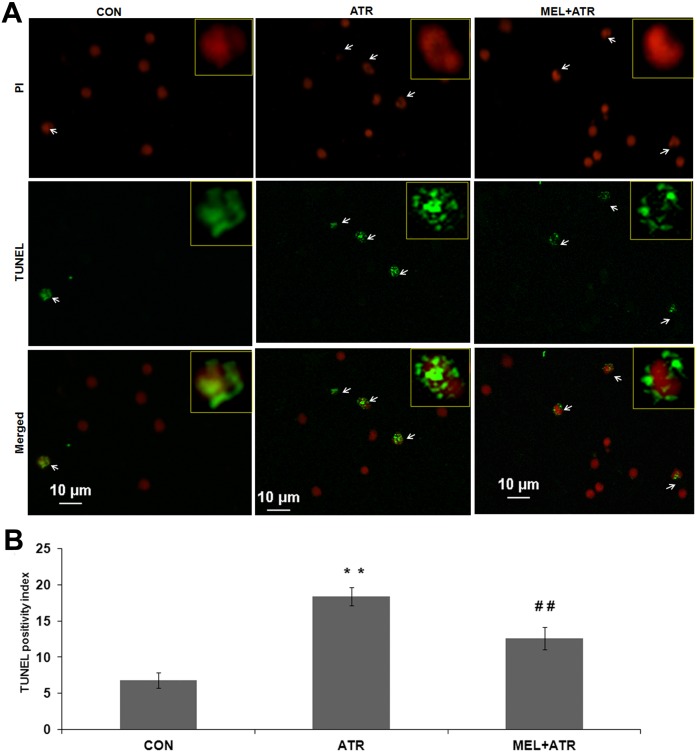
ATR-induced apoptosis in murine splenocytes was ameliorated by MEL. (A) Representative images of TUNEL staining in splenocytes of control (CON), atrazine (ATR) and melatonin with atrazine co-treated (MEL+ATR) mice. All cell nuclei were stained with propidium iodide (red fluorescence). TUNEL (fluorescein-12-dUTP labeled fragmented DNA) positive nuclei, displaying green fluorescence, are arrow-marked. Insets show individual and merged fluorescence of single cells at a higher magnification. (B) Histogram showing TUNEL positivity index. Data are expressed as mean ± SEM of 3 experiments (**P<0.01 versus CON; ^##^ *P*<0.01 versus ATR).

The binding of Annexin V-FITC to phosphatidylserine on outer leaflet of cell membrane, which is an early morphological event of apoptotic cells, was detected by flow cytometry. FITC+/PI- single green fluorescence was located in lower right quadrants (Figure S2, a). ATR treatment significantly enhanced (*P*<0.01 versus CON) the apoptotic index, whereas MEL co-treatment led to its significant reduction (*P*<0.01 versus ATR; Figure S2, b). DNA fragmentation was confirmed by TUNEL assay wherein apoptotic cells display green nuclei (marked with arrows; Fig. 1A). ATR treatment produced a significant increase (*P*<0.01 versus CON) in TUNEL positivity which was reduced significantly by MEL (*P*<0.01 versus ATR; Fig. 1B). Altogether these results show that ATR induced apoptosis in splenocytes, which was reversed by MEL.

### Fas mediated apoptosis in splenocytes induced by ATR was inhibited by MEL

**Figure 2:**
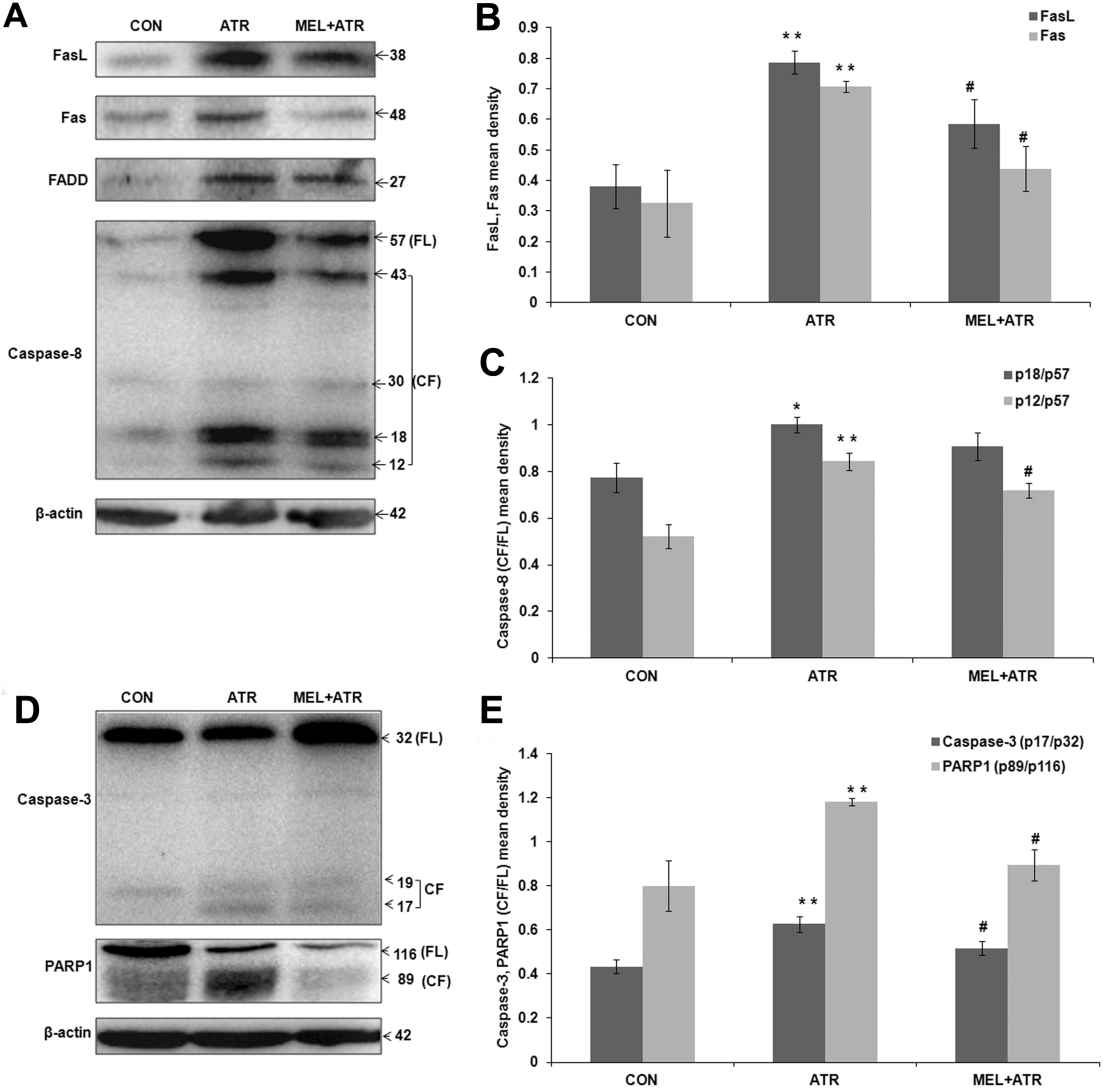
ATR-induced Fas mediated apoptosis was inhibited by MEL. (A) Representative immunoblots of splenocyte lysates from CON, ATR and MEL+ATR mice. FasL, Fas, FADD, Caspase-8 and β-actin proteins were visualized by chemiluminiscence. Corresponding histograms show (B) FasL, Fas and (C) Caspase-8 (CF/FL; p18/p57 and p12/p57) expressions as mean densities normalized by β-actin density. (D) Representative immunoblots of Caspase-3, PARP1 and β-actin. Corresponding histogram (E) shows caspase-3 (CF/FL, p17/p32) and PARP1 (CF/FL, p89/p116) mean densities normalized by β-actin. Data are presented as mean ± SEM of 3 independent experiments (*P<0.05, **P<0.01 versus CON; ^#^ *P*<0.05 versus ATR). FL, full length; CF, cleaved fragments.

Expressions of regulatory proteins of the death receptor family were quantified by immunoblot assay (Fig. 2A). Significant enhancement in expressions of Fas ligand (FasL) and Fas (*P*<0.01 versus CON; Fig. 2B) were accompanied by an increase in FADD (Fas-associated protein with death domain) (Fig. 2A). This led to autocatalytic cleavage of inactive full length caspase-8 (57 kDa), a downstream target of Fas pathway, into its 18 and 12 kDa active fragments (p18/p57; *P*<0.05 and p12/p57; *P*<0.01, respectively, versus CON; Fig. 2C). MEL co-treatment significantly inhibited the FasL and Fas expressions (*P*<0.05 versus ATR; Fig. 2B), accompanied by suppression of caspase-8 activation, as demonstrated by a decline in the ratio of cleaved to full length protein (p12/p57; *P*<0.05 versus ATR; Fig. 2C).

The Fas-mediated, Caspase-8 dependent pathway led to activation of effector caspase-3 followed by cleavage of its downstream target PARP1 (Fig. 2D) which execute DNA fragmentation. ATR treatment resulted in activation of caspase-3, releasing its 17 kDa active fragment (p17/p32; *P*<0.01 versus CON; Fig. 2E) which further cleaved full length PARP1 (116 kDa) to its inactive 89 kDa fragment (p89/p116; *P*<0.01 versus CON; Fig. 2E), thereby attenuating the DNA repair function of PARP1 [31]. MEL co-treatment resulted in inhibition of ATR-induced DNA fragmentation, as evident from suppression of the cleavage of caspase-3 (p17/p32; *P*<0.05 versus ATR) and PARP1 (p89/p116; *P*<0.05 versus ATR; Fig. 2E).

### Mitochondrial apoptosis induced by ATR was inhibited by MEL

**Figure 3:**
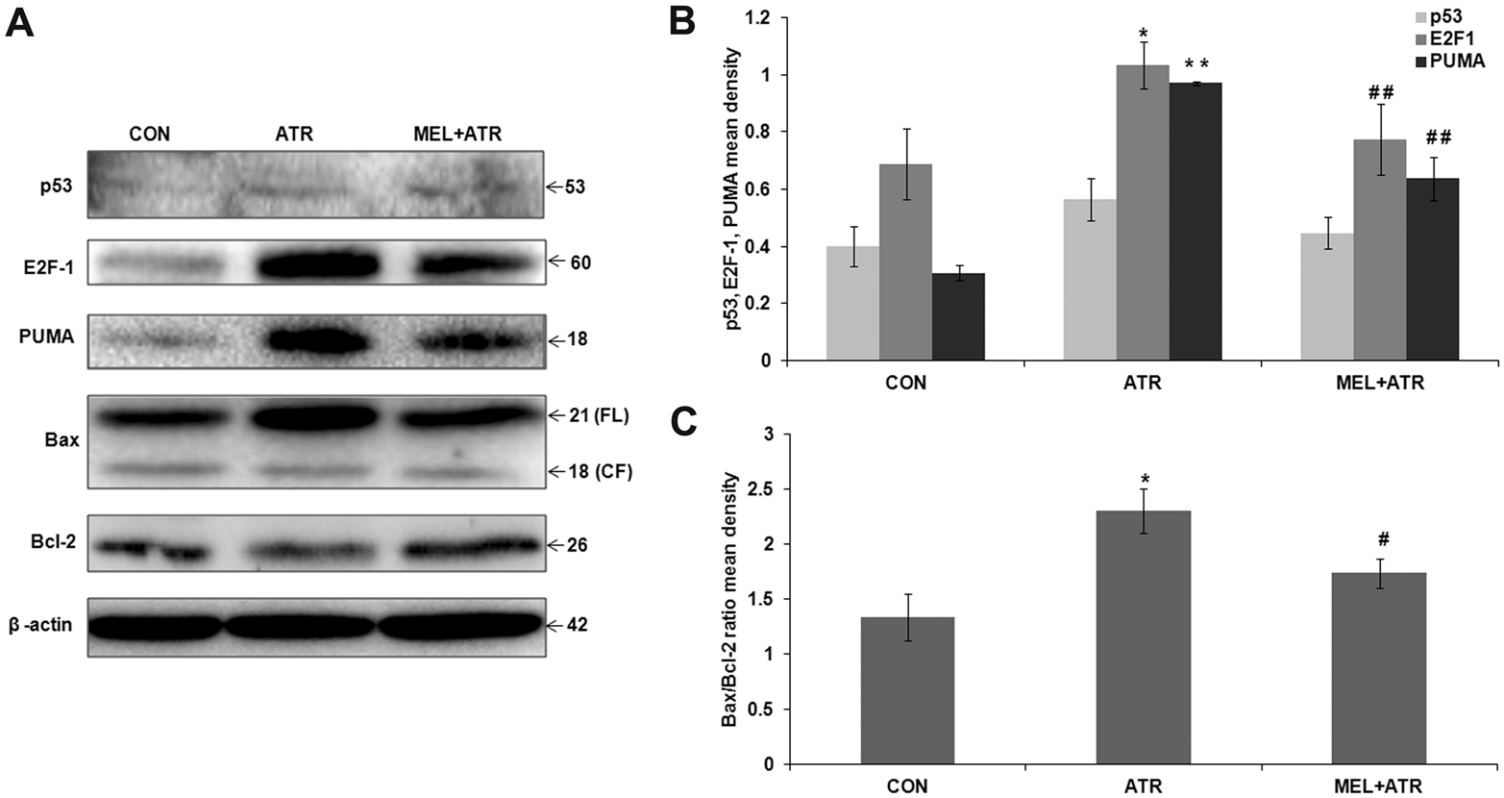
ATR-induced mitochondria mediated apoptosis was inhibited by MEL. (A) Representative immunoblots of p53, E2F-1, PUMA, Bax, Bcl-2 and β-actin in CON, ATR and MEL+ATR groups. Histograms show (B) p53, E2F-1, PUMA and (C) Bax/Bcl-2 ratio mean densities normalized by β-actin density. Data are expressed as mean ± SEM of 3 independent experiments (*P<0.05, **P<0.01 versus CON; ^#^ *P*<0.05, ^##^ *P*<0.01 versus ATR).

To learn whether ATR treatment also induces mitochondrial apoptosis in splenocytes, we investigated the expressions of p53, E2F-1, PUMA, Bax and Bcl-2 proteins which regulate mitochondria-mediated apoptosis [32], [33]. ATR treatment significantly enhanced the expressions of E2F-1 (*P*<0.05 versus CON) and PUMA (*P*<0.01 versus CON), though p53 level remained unaltered when compared with control (Fig. 3A and 3B). Further, E2F-1 and PUMA initiate mitochondrial apoptosis by regulating their downstream targets Bax and Bcl-2. We observed a significant increase in Bax/Bcl-2 ratio (*P*<0.05 versus CON; Fig. 3C) upon ATR treatment. Co-treatment with MEL did also not alter the p53 level (Fig. 3B). However, it suppressed E2F-1 and PUMA expressions (*P*<0.01 versus ATR; Fig. 3B) as well as Bax/Bcl-2 ratio (*P*<0.05 versus ATR; Fig. 3C) thereby inhibiting the mitochondria-mediated apoptosis induced by ATR.

### ER stress induced by ATR was inhibited by MEL

**Figure 4:**
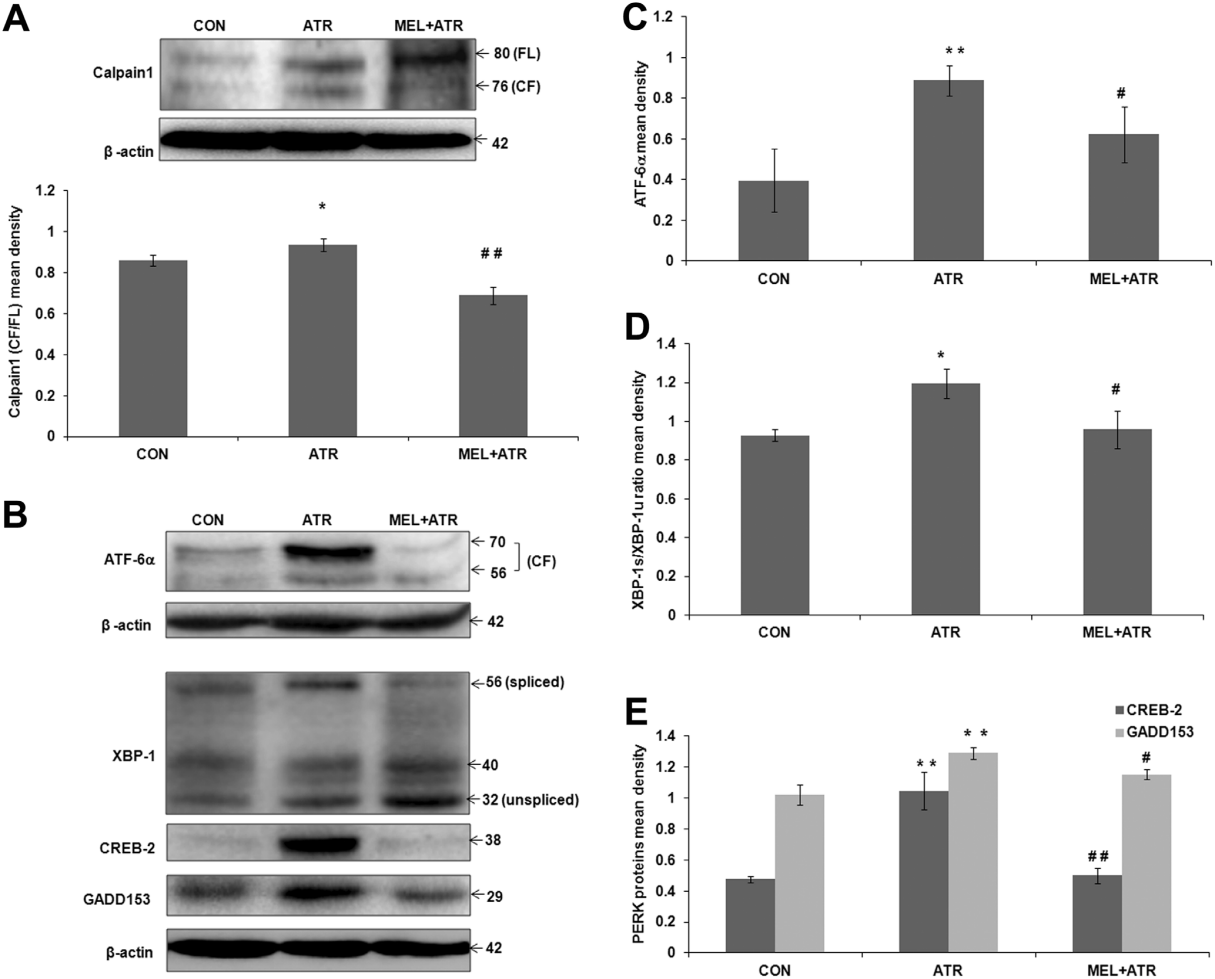
ATR-induced ER stress response in splenocytes was ameliorated by MEL. (A) Representative immunoblot showing Calpain1 cleavage and histogram showing ratio of active to inactive Calpain1 (CF/FL, p76/p80) mean density normalized by β-actin. (B) Representative immunoblots of ATF6α, XBP-1, CREB-2, GADD153 and β-actin. Histograms show mean densities of (C) ATF-6α (CF, 70), (D) XBP-1s/XBP-1u ratio (56/32), and (E) PERK proteins (CREB-2 and GADD153) normalized by β-actin density. Data are expressed as mean ± SEM of 3 independent experiments (*P<0.05, **P<0.01 versus CON; ^#^ *P*<0.05, ^##^ *P*<0.01 versus ATR).

Along with expression of full length Bax (21 kDa), we also noted the occurrence of its cleaved 18 kDa fragment, even though p18 expression levels remained constant (Fig. 3A). Since p18 is known to be released in response to Calpain1 activation, we went on to check for Calpain1 expression as a marker for ER stress [34]. ATR treatment resulted in cleavage of inactive Calpain1 (80 kDa) to its catalytically active 76 kDa fragment (p76/p80; *P*<0.05 versus CON). This cleavage was significantly inhibited by MEL co-treatment (*P*<0.01 versus ATR; Fig. 4A).

Since Calpain1 activation also suggested the involvement of ER stress in induction of apoptosis, we performed immunostaining of marker proteins for each of the three branches of ER stress pathway: ATF-6α (for ATF6 branch), XBP-1s (for IRE1 branch) and GADD153 (for PERK branch). Signal positive cells were observed in ATR as well as MEL co-treated groups (Figure S3). Further, we quantified the expression levels of these ER stress markers by immunoblot assays (Fig. 4B). ATR treatment caused excessive ER stress, as evident from the activation of ATF-6α (*P*<0.01 versus CON; Fig. 4C) and overexpression of XBP-1s (p56/p32; *P*<0.05 versus CON; Fig. 4D), CREB-2 and GADD153 proteins (*P*<0.01 versus CON; Fig. 4E). MEL co-treatment, on the other hand, inhibited the formation of ATF-6α (*P*<0.05 versus ATR; Fig. 4C) and reduced the XBP-1s/XBP-1u ratio (*P*<0.05 versus ATR; Fig. 4D). It also downregulated CREB-2 expression, followed by inhibition of the expression of its downstream target GADD153 (*P*<0.01 and *P*<0.05, respectively, versus ATR; Fig. 4E). These results reflected that MEL is capable of suppressing all three branches of ER stress response generated by ATR in splenocytes.

### ATR-mediated impairment of autophagy was ameliorated by MEL

**Figure 5:**
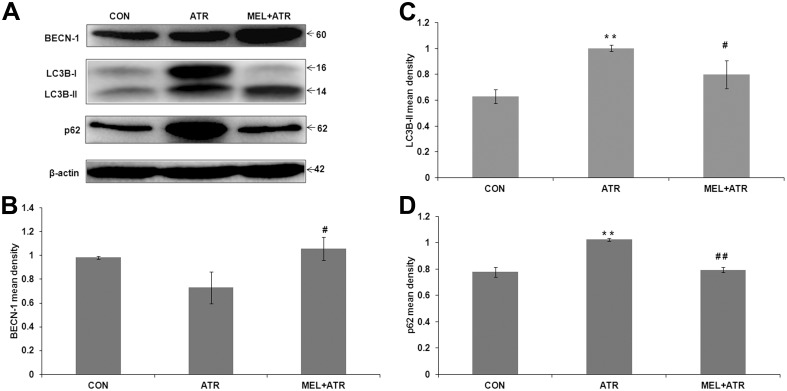
ATR-induced dysregulation of autophagy in splenocytes was ameliorated by MEL. (A) Representative immunoblots of autophagy markers BECN-1, LC3B (I and II), p62 and loading control β-actin in CON, ATR and MEL+ATR groups. Histograms show (B) BECN-1, (C) LC3B-II and (D) p62 mean densities normalized by β-actin. Data are presented as mean ± SEM of 3 experiments (**P<0.01 versus CON; ^#^ *P*<0.05, ^##^ *P*<0.01 versus ATR).

Autophagy plays an important role in the recovery from ER stress [17] hence we investigated whether ATR and MEL could also modulate the key autophagic signals BECN-1, LC3B-II and p62/SQSTM1. BECN-1 initiates the formation of autophagosomes whereupon LC3B-II gets recruited. LC3B-II, which remains localized to autophagic structures throughout the process of autophagy, binds to p62 laden with the cargo of poly-ubiquitinated proteins and targets the cargo (together with p62) for degradation at the autolysosome. Thus, elevated levels of BECN-1 and LC3B-II and a reduced level of p62 proteins signify an effective autophagy process [35]. However, presently we saw downmodulation of BECN-1 (versus CON, though statistically not significant; Fig. 5A and 5B) and enhancement of LC3B-II (*P*<0.01 versus CON; Fig. 5A and 5C) as well as p62 (*P*<0.01 versus CON; Fig. 5A and 5D) expressions in splenocytes following ATR treatment suggesting an impairment of autophagy. Co-treatment with MEL ameliorated this impairment by way of a significantly elevated expression of BECN-1 (*P*<0.05 versus ATR; Fig. 5B) along with decline in LC3B-II and p62 levels (*P*<0.05 and *P*<0.01, respectively, versus ATR; Fig. 5C and 5D).

## Discussion

In this study, we aimed at elucidating the apoptotic mechanisms contributing to immunotoxicity of the herbicide Atrazine and exploring whether and how Melatonin could attenuate them. The short-course treatment of mice with ATR resulted in apoptosis of splenocytes as evident from the results of Annexin V binding and TUNEL assays. An enhanced expression of FasL and Fas in these cells suggested activation of the Fas mediated pathway, as also reported previously [13]. The FasL-Fas interaction further led to DISC formation with recruitment of FADD and procaspase-8, culminating in the release of active fragments of caspase-8 (p43, p30, p18 and p12). Caspase-8 activation at DISC is a complex process occurring via autocatalysis. During the first cleavage, p43 and p30 products are released which further generate their active fragments p12 and p18, respectively [36]. ATR treatment significantly enhanced expression of both the fragments. The active caspase-8 cleaves caspase-3 which executes DNA fragmentation [36]. In addition, the ATR-induced inactivation of PARP1, which plays a role in DNA repair [31], could also have contributed to apoptosis.

Induction of the mitochondria-mediated pathway of apoptosis by ATR was indicated by an increase in the ratio of proapoptotic Bax to antiapoptotic Bcl-2 proteins. Increase in Bax/Bcl-2 ratio suggests translocation of Bax to mitochondria, permeabilization of mitochondrial membrane and activation of caspases. Mitochondria-mediated apoptosis proceeds primarily through activation of the transcription factor p53 which further activates its downstream targets Bax, PUMA (p53 upregulated modulator of apoptosis) and represses Bcl-2 [33]. The p53 dependent mitochondrial apoptosis has previously been reported in case of herbicides [37]. However, presently a uniformly low expression of p53 in control as well as experimental groups suggested induction of apoptosis through the p53 independent pathway involving E2F-1. E2F-1 is a transcription factor of E2F family having binding sites within the promoters of p53, Puma, Apaf-1, p73 and GRAMD4. Hence it is capable of causing mitochondrial apoptosis through p53 dependent as well as independent mechanisms [32], [38]. Both E2F-1 and PUMA proteins were overexpressed after treatment with ATR. This, to our knowledge, is the first documentation of p53 independent mitochondrial apoptosis as a mechanism for toxicity of herbicides.

The 18 kDa cleaved fragment (p18) of Bax was seen invariably in control as well as experimental splenocytes. Though it appeared as a faint band due perhaps to a short half-life (2 h) [39], its occurrence prompted us to look for Calpain1 activity which is known to cleave Bax (21 kDa) into its p18 fragment [34]. Importantly, overexpression of Calpain1 in response to ATR treatment also suggested the involvement of ER stress pathway in induction of apoptosis [37]. ER stores Ca^2+^ and maintains Ca^2+^ homeostasis for optimal protein folding. Under conditions of stress, Ca^2+^ is released from the lumen of ER into cytosol and triggers activation of Calpains [40] leading to activation of one or more of the three branches of ER stress. Activation of ATF6 branch causes production of the cleaved product ATF-6α (56 kDa, 70 kDa) which induces transcription of XBP-1u mRNA. Under the IRE1 branch, XBP-1u mRNA is processed by IRE1 to generate its spliced form XBP-1s (56 kDa) which upregulates ER chaperones and ERAD (endoplasmic reticulum stress associated protein degradation) components [41]. In case of mammals, XBP-1u mRNA also gets translated to the corresponding protein (32 kDa) in cytosol [42]. The PERK branch of ER stress involves activations of CREB-2 followed by GADD153 which attenuate protein translation [43]. As evident from the expression profile of target proteins (cleaved ATF-6α, XBP-1s, CREB-2 and GADD153), ATR treatment activated all three branches of ER stress. Though ER stress normally helps in cell survival by removing misfolded proteins, an elevated and prolonged ER stress level can cause apoptosis [14]. Presently, ER stress-mediated apoptosis induced by ATR was evident from overexpression of the proapoptotic signals GADD153 and PUMA. GADD153 is known to bind to Puma promoter and transactivate PUMA expression [44]. In a related study, the herbicide arsenite has also been shown to induce ER stress and apoptosis [23].

Autophagy serves to ameliorate the ER stress in normal cells [17]. Onset of autophagy is signalled by BECN-1, which forms a ‘core complex’ (BECN1-Vps34-Vps15) that gets localized on the ‘pre-autophagosomal’ structures [45]. LC3B-II gets integrated into autophagosomes and selects the cargo of p62-associated protein aggregates for degradation by autolysosomes. Thus enhanced LC3B-II, together with reduced p62 levels are hallmarks of efficient autophagy [35]. However, expression levels of both markers were enhanced in splenocytes after ATR treatment, suggesting an impairment of autophagy. A possible reason for this impairment could be the low expression level of BECN-1which resulted in a low turnover of autophagosomes leading to accumulation of LC3B-II and p62. In addition, ATR-induced caspases 3 and 8 (discussed above) could also have degraded BECN-1 and caused its paucity in splenocytes [46]. Alternatively, a ‘block’ in autophagic flux at the level of autolysosome generation or activation could also cause accumulation of LC3B-II and p62 [47], [48]. Though such a possibility can be explored with the help of specific inhibitors [35], there are certain caveats to this approach, particularly when applied to *in* *vivo* studies. As the rate of basal autophagic flux for most tissues is unknown, short treatments with the inhibitors may not be effective and long treatments could produce toxicity [49], [50]. Moreover, protracted treatments with inhibitors can also lead to ‘off-target’ effects [35]. For example, 3-methyladenine (3-MA) can, in long term experiments, promote autophagy as well as reduce cell survival.

Melatonin is known to regulate oxidative stress, apoptosis and mitochondrial homeostasis through its free radical scavenging action and interaction with receptors and intracellular targets involved in signal transduction [51]. The protective action of MEL against the death receptor as well as mitochondria-mediated apoptosis induced by ATR in splenocytes was evident from inhibition of FasL, Fas, FADD, caspase-8 and suppression of Bax/Bcl-2 ratio. Suppression of caspase-8 activity through Fas pathway provides a new insight into the cytoprotective action of MEL. In an earlier study, MEL was found to abrogate caspase-8 activity in rabbit liver by modulating the TNF-mediated (rather than Fas-mediated) pathway [24]. MEL is also known to manifest its antiapoptotic effect through suppression of p53 dependent mitochondrial apoptosis [52]. However, the p53 independent (E2F-1 and PUMA dependent) pathway [32], [53] was apparently involved in the present case. In addition, cytoprotective action of MEL also involved suppression of caspase-3 cleavage and activation of PARP1 as reported previously [24].

MEL appeared to protect against ER stress through inhibition of Calpain1 activation. Samantaray *et al.* [54] have also demonstrated that MEL attenuates activation of both Calpain and caspase-3 in spinal cord injury of rats. In addition, MEL co-treatment attenuated the activation of ER stress markers ATF-6α, XBP-1s, CREB-2 and GADD153 suggesting suppression of all three branches of ER stress which were activated by ATR. In a relevant report, Zhao *et al.* [55] have also demonstrated downmodulation of the three branches of ER stress by MEL in the mouse model of bleomycin-induced pulmonary fibrosis. During recovery from ER stress, the expression of XBP-1u protein is enhanced which acts as a feedback inhibitor for XBP-1s protein [56]. Thus, the increased expression of XBP-1u in response to MEL co-treatment suggested ER stress recovery in splenocytes.

Enhancement of autophagy signaling in splenocytes after MEL co-treatment was evident from a significant increase in BECN-1 and decrease in LC3B-II and p62 expression levels (compared with levels after ATR treatment). In particular, the decrease in p62 expression, which is a key indicator of normal autophagy process [26], signalled recovery from the ATR-induced dysregulation of autophagy. The downmodulation of caspases by MEL could also have contributed to this recovery by preventing the caspase-mediated degradation of BECN-1 [47]. Though we have not looked for it, MEL could possibly have favourably modulated certain other components of the autophagy flux. For instance, in case of ‘cytoprotective autophagy’ the cleaved 43 kDa fragment of caspase-8 co-localizes with LC3B-II and gets degraded by autolysosomes [57]. This could have been the reason behind the observed low expression of p12 (a product of p43) in the MEL co-treated group.

**Figure 6:**
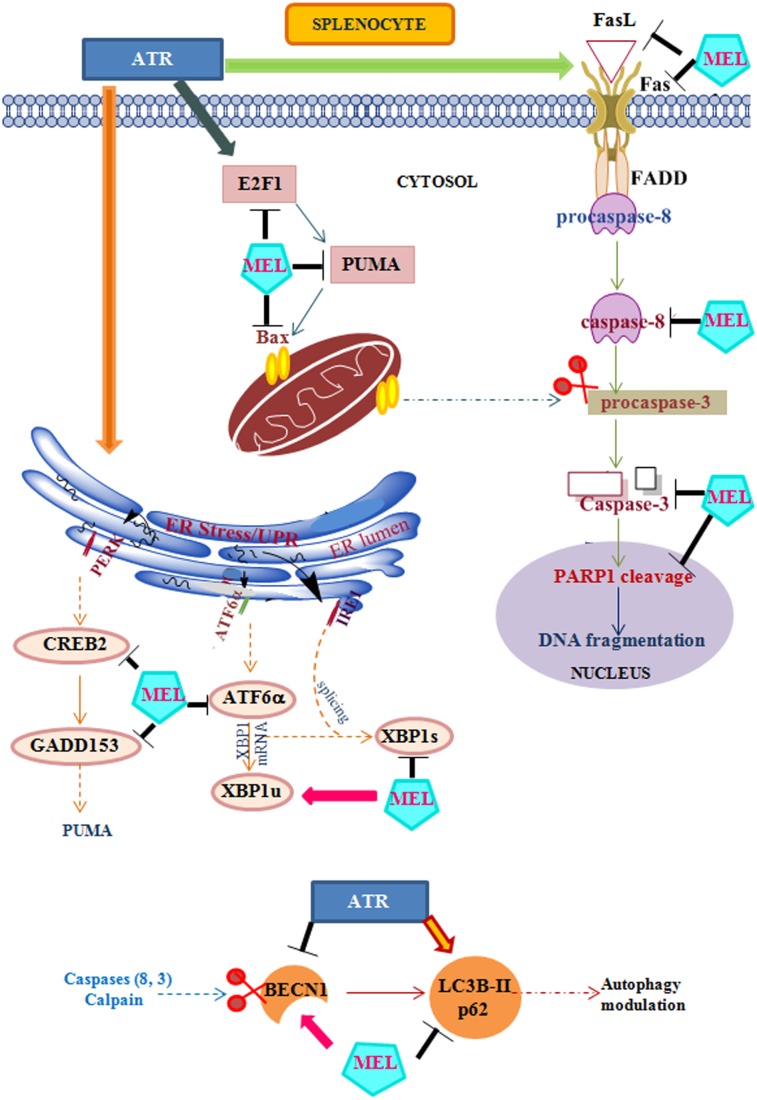
Schematic diagram showing protective action of MEL against ATR immunotoxicity. ATR treatment activates death receptor (FasL, Fas, FADD, Caspase-8) and mitochondrial (E2F-1, PUMA, Bax) apoptosis (Caspase-3 and cleaved PARP1) signals. In addition, ATR induces ER stress (ATF-6α, XBP-1s, CREB-2, GADD153) signals. MEL inhibits the Fas and mitochondrial apoptosis as well as ER stress. ATR treatment also impairs autophagy by suppressing BECN-1 and upregulating LC3B-II and p62 proteins; whereas MEL restores autophagy by reversing this dysregulation. Dotted line arrows indicate known connecting pathways that were not a part of the present study. Line arrows indicate stimulatory effect and sign T indicates inhibitory effect on the expression of corresponding proteins. Scissor symbol indicates the cleavage of target proteins.

We have summarized our results in the form of a diagrammatic pathway (Fig. 6). ATR triggers the death receptor (Fas) mediated as well as mitochondrial (E2F-1, PUMA, Bax) apoptosis in mouse splenocytes. It also induces an excessive ER stress response, as evident from activation of ATF-6α (ATF6 branch), XBP-1s (IRE1 branch), CREB-2 and GADD153 (PERK branch). Further, it dysregulates the expression of key autophagy signals BECN-1, LC3B-II and p62. Exogenous MEL, on the other hand, attenuates both Fas-mediated and E2F-1 dependent mitochondria- mediated apoptosis, suppress all three branches of ER stress and enhances autophagy to protect the splenocytes.

In conclusion, our study highlights multiple targets of cell death which could play a role in immunotoxicity of ATR. MEL imparted protection against this apoptosis through, hitherto unreported, ‘Fas-mediated’ and ‘p53 independent E2F-1 dependent’ pathways. The latter finding opens a possibility that some other p53 independent pathways (such as p73 and Noxa) could also be involved in anti- apoptotic activity of MEL. MEL also protected splenocytes from ATR-induced ER stress and promoted autophagy. A concerted regulation of ER stress, apoptosis and autophagy targets by exogenous MEL in this manner adds a new dimension to its protective role against immunotoxicity.

## Supporting Information

Figure S1 **ATR-induced toxicity in lymphoid organs (spleen and thymus) of mice and its reversal by MEL.** Histogram shows effect of ATR and MEL treatments on relative spleen and thymus weights (CON, control; ATR, atrazine; MEL+ATR, melatonin and atrazine co-treated group). Data are expressed as mean ± SEM (n = 6) (*P<0.05, **P<0.01 versus CON; ^#^ *P*<0.05 versus ATR).
(TIF)

Figure S2 **MEL inhibited ATR induced early apoptosis.** (a) Representative dot plots of Annexin V-FITC and PI stained apoptotic cells were analyzed by flow cytometry in CON, ATR and MEL+ATR groups. Lower right quadrant displays apoptotic cells with FITC+/PI- stains. (b) Histogram shows apoptotic index. Data are expressed as mean ± SEM of 3 experiments (**P<0.01 versus CON; ^##^ *P*<0.01 versus ATR).
(TIF)

Figure S3 **Effect of ATR and MEL on ER stress response in splenocytes.** Representative photomicrographs show immunoreactivity of (a) ATF-6α of ATF6 branch, (b) XBP-1 of IRE1 branch and (c) GADD153 of PERK branch. Nuclei stained with DAPI fluoresced blue (Lane 1), cells expressing protein signals fluoresced red (white arrow, Lane 2). Lane 3 shows merged photomicrographs (Scale bar = 10 µM).
(TIF)

Table S1 Sources and dilutions of antibodies used for immunoblot (IB) and immunofluorescence (IF) assays.
(DOCX)
