# Supplementary figures and images for: Melatonin Reverses Fas, E2F-1 and Endoplasmic Reticulum Stress Mediated Apoptosis and Dysregulation of Autophagy Induced by the Herbicide Atrazine in Murine Splenocytes
Source: PLoS One. 2014 Sep 26;9(9):e108602. doi: 10.1371/journal.pone.0108602 (PMC4178181; doi:10.1371/journal.pone.0108602)

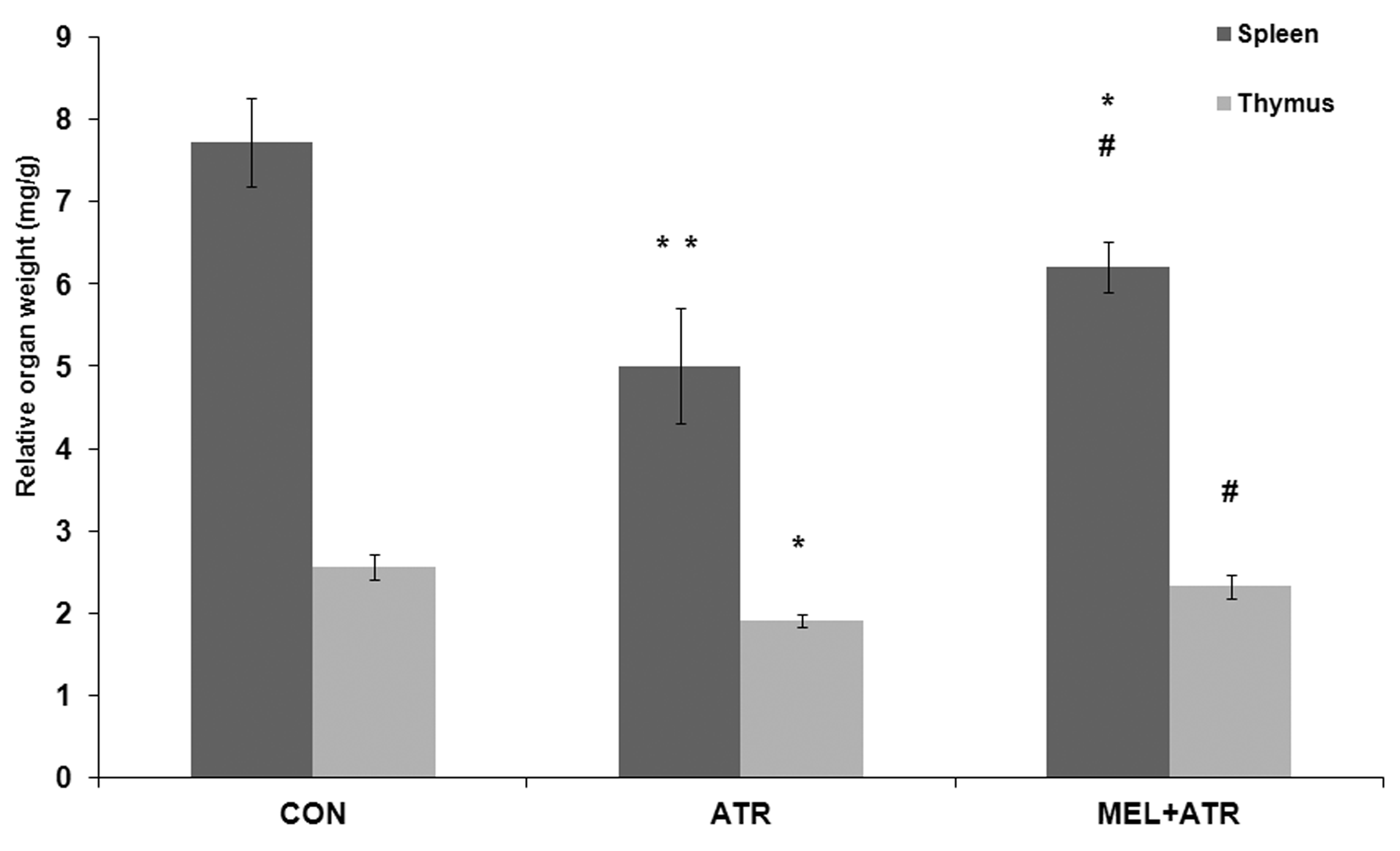

Supplement: Figure S1 — ATR-induced toxicity in lymphoid organs (spleen and thymus) of mice and its reversal by MEL. Histogram shows effect of ATR and MEL treatments on relative spleen and thymus weights (CON, control; ATR, atrazine; MEL+ATR, melatonin and atrazine co-treated group). Data are expressed as mean ± SEM (n = 6) (*P<0.05, **P<0.01 versus CON; # P<0.05 versus ATR). (TIF) [file pone.0108602.s001.tif]

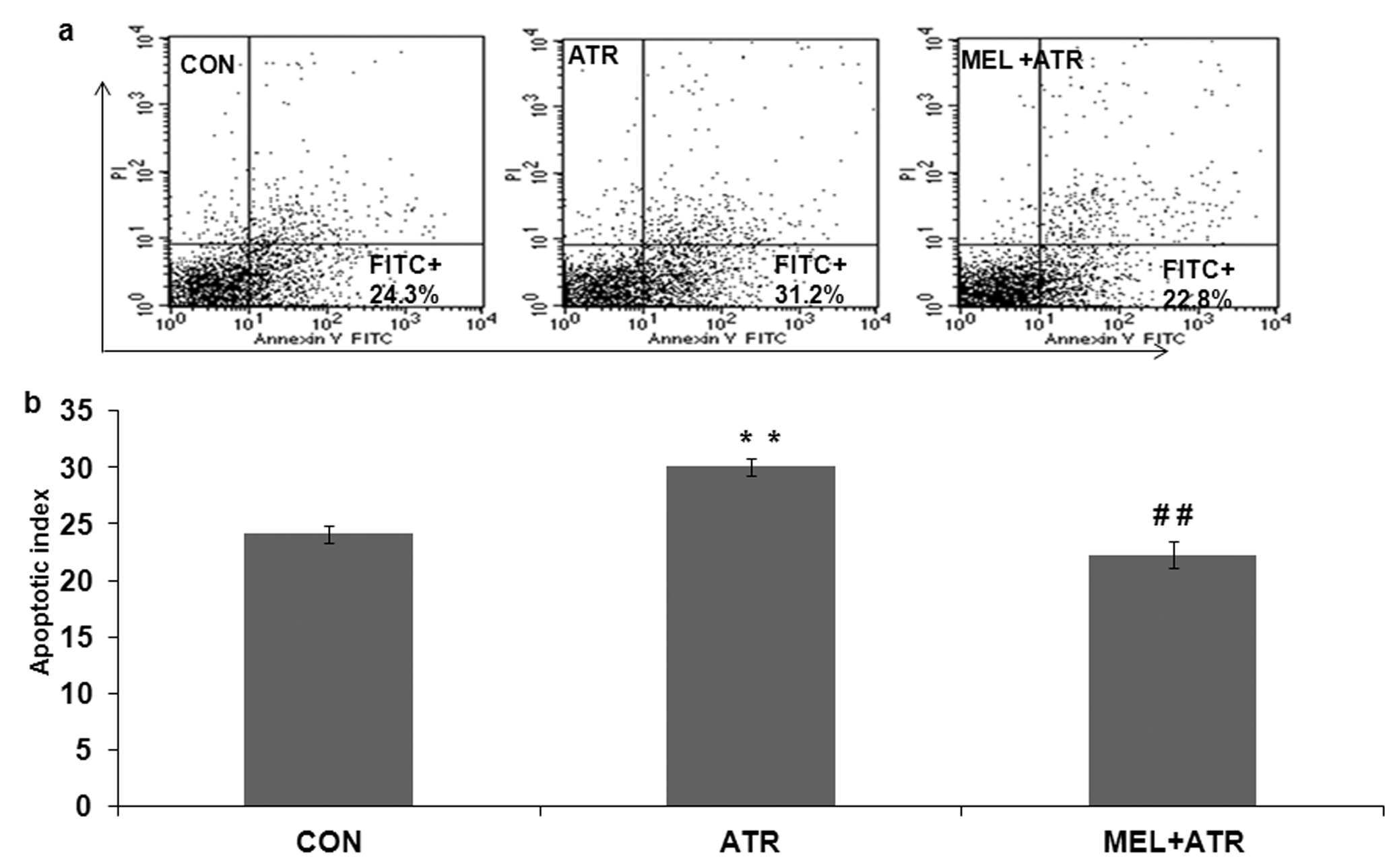

Supplement: Figure S2 — MEL inhibited ATR induced early apoptosis. (a) Representative dot plots of Annexin V-FITC and PI stained apoptotic cells were analyzed by flow cytometry in CON, ATR and MEL+ATR groups. Lower right quadrant displays apoptotic cells with FITC+/PI- stains. (b) Histogram shows apoptotic index. Data are expressed as mean ± SEM of 3 experiments (**P<0.01 versus CON; ## P<0.01 versus ATR). (TIF) [file pone.0108602.s002.tif]

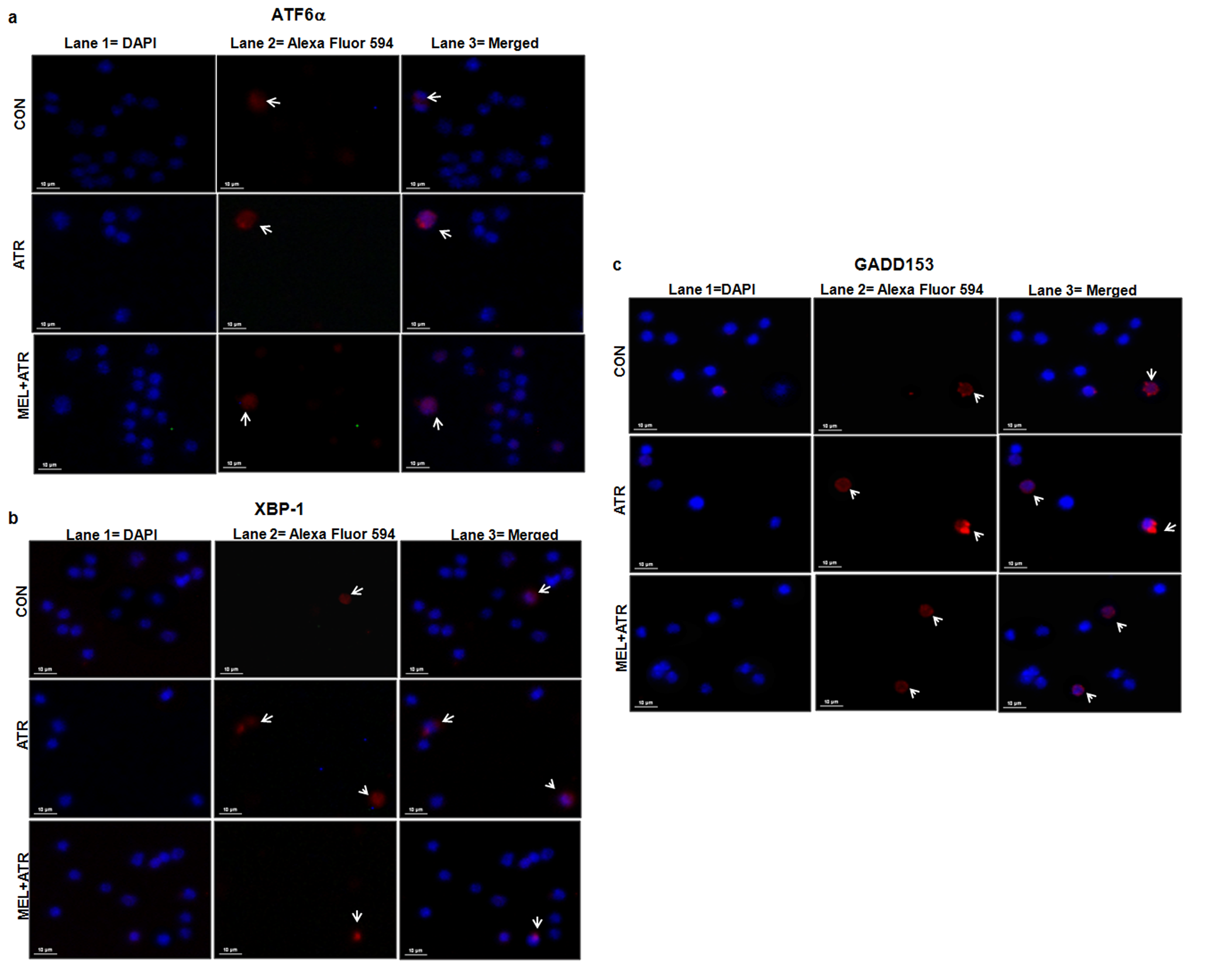

Supplement: Figure S3 — Effect of ATR and MEL on ER stress response in splenocytes. Representative photomicrographs show immunoreactivity of (a) ATF-6α of ATF6 branch, (b) XBP-1 of IRE1 branch and (c) GADD153 of PERK branch. Nuclei stained with DAPI fluoresced blue (Lane 1), cells expressing protein signals fluoresced red (white arrow, Lane 2). Lane 3 shows merged photomicrographs (Scale bar = 10 µM). (TIF) [file pone.0108602.s003.tif]
