# Supplementary material for: Melatonin Reverses Fas, E2F-1 and Endoplasmic Reticulum Stress Mediated Apoptosis and Dysregulation of Autophagy Induced by the Herbicide Atrazine in Murine Splenocytes
Source: PLoS One. 2014 Sep 26;9(9):e108602. doi: 10.1371/journal.pone.0108602 (PMC4178181; doi:10.1371/journal.pone.0108602)
Supplement: Table S1 — Sources and dilutions of antibodies used for immunoblot (IB) and immunofluorescence (IF) assays. (DOCX) [file pone.0108602.s004.docx]

**Table S1. Sources and dilutions of antibodies used for immunoblot (IB) and immunofluorescence (IF) assays.**

| **S/n** | **Antibody** | **Dilution** | **Host** | **Company** |
| --- | --- | --- | --- | --- |
| 1. | Bax (P-19) | 1:500 (IB) | Rabbit | Santa Cruz |
| 2. | Bcl-2 (N-19) | 1:400 (IB) | Rabbit | Santa Cruz |
| 3. | Fas (A-20) | 1:400 (IB) | Rabbit | Santa Cruz |
| 4. | FasL (N-20) | 1:400 (IB) | Rabbit | Santa Cruz |
| 5. | FADD (H-181) | 1:350 (IB) | Rabbit | Santa Cruz |
| 6. | Caspase-8 (4790) | 1:1000 (IB) | Rabbit | Cell Signaling |
| 7. | Caspase-3 ((H-277) | 1:400 (IB) | Rabbit | Santa Cruz |
| 8. | PARP1 (9542) | 1:1000 (IB) | Rabbit | Cell Signaling |
| 9. | E2F-1 (KH95) | 1:300 (IB) | Mouse | Santa Cruz |
| 10. | p53 (FL-393) | 1:500 (IB) | Rabbit | Santa Cruz |
| 11. | PUMA (7467) | 1:1000 (IB) | Rabbit | Cell Signaling |
| 12. | Calpain-1 (N-19) | 1:350 (IB) | Goat | Santa Cruz |
| 13. | ATF-6α (H-280) | 1:300 (IB); 1:50 (IF) | Rabbit | Santa Cruz |
| 14. | XBP-1 (M-186) | 1:300 (IB); 1:50 (IF) | Rabbit | Santa Cruz |
| 15. | CREB-2 (C-20) | 1:350 (IB) | Rabbit | Santa Cruz |
| 16. | GADD153 (B-3) | 1:350 (IB); 1:50(IF) | Mouse | Santa Cruz |
| 17. | BECN-1 (D-18) | 1:700 (IB) | Goat | Santa Cruz |
| 18. | LC3B (L7543) | 1 µg/ml (IB) | Rabbit | Sigma Aldrich |
| 19. | p62 (P0067) | 0.75 µg/ml (IB) | Rabbit | Sigma Aldrich |
| 20. | β-actin (A2228) | 1:5000 (IB) | Mouse | Sigma Aldrich |
